# Clinical outcomes and survival following lung transplantation for work-related lung disease: a single-center retrospective cohort study

**DOI:** 10.1186/s12995-023-00368-4

**Published:** 2023-02-13

**Authors:** Chunrong Ju, Yalan Yang, Qiaoyan Lian, Lulin Wang, Xiaohua Wang, Bing Wei, Danxia Huang, Xin Xu, Jianxing He

**Affiliations:** State Key Laboratory of Respiratory Disease, National Clinical Research Center for Respiratory Disease, Guangzhou Institute of Respiratory Health, First Affiliated Hospital of Guangzhou Medical University, Guangzhou, 510120 China

**Keywords:** Work-related lung disease, Lung transplantation, Idiopathic pulmonary fibrosis, Pneumothorax, Survival rate

## Abstract

**Background:**

Patients with work-related lung disease (WRLD) are at increased risk of death caused by severe lung tissue damage and fibrosis. This study aimed to assess the clinical outcomes of lung transplantation (LTx) for WRLD and compare the results of LTx between WRLD and idiopathic pulmonary fibrosis (IPF).

**Methods:**

This single-center retrospective cohort study reviewed the clinical data of patients who underwent LTx for WRLD or IPF at our hospital between January 2015 and December 2021. Cumulative survival rates after LTx were estimated using the Kaplan-Meier method.

**Results:**

The final analysis included 33 cases of WRLD and 91 cases of IPF. The 33 WRLD patients consisted of 19 (57.6%) cases of silicosis, 8 (24.2%) cases of coal workers’ pneumoconiosis, 3 (9.09%) cases of asbestosis, and 3 (9.09%) cases of other WRLD. Pneumothorax as an indication for LTx was significantly more common in the WRLD group than in the IPF group (51.5% vs. 2.2%, *P* < 0.001). There was no significant difference in the 5-year cumulative survival rate between the WRLD patients and the IPF patients (66.6% vs. 56.7%, *P* = 0.67). There was no significant difference in the best performance of exercise capacity and lung function between the two groups at 1 year post-transplant.

**Conclusions:**

LTx had similar survival outcomes and lung function for WRLD and IPF patients. Pneumothorax was the primary indication for lung transplantation in WRLD.

## Background

Work-related lung disease (WRLD) refers to a spectrum of pulmonary diseases caused by inhalation of mineral dusts and the lung tissue reactions, which eventually induces irreversible lung damage and fibrotic lung diseases, leading to lung dysfunction and respiratory failure. The most common types of WRLD include silicosis, coal workers’ pneumoconiosis, asbestosis, metal pneumoconiosis, and berylliosis. The global prevalence of WRLD is around 527,500 cases, with over 60,000 new cases reported in 2017 [1]. The mortality of WRLD remains at a high level in recent years, with over 21,000 deaths [2]. Alarmingly, WRLD has re-emerged even in some developed countries, and its prevalence in developing countries is even higher [3, 4]. It is also the most common occupational disease in China [5]. The number of patients with WRLD in China has increased significantly in recent years with 0.87 million cumulative cases until 2018.

WRLD is not only a potential cause of disability but also a leading cause of death in occupational diseases in China [6]. Studies during the last decade have failed to find an effective cure for WRLD. Thus, lung transplantation (LTx) becomes the only effective therapeutic option for end-stage WRLD. However, this procedure requires significant social support and is a financial burden on patients and their families, which can be prohibitive for placing a potential WRLD recipient on the transplant waitlist. Also, there is still a paucity of data regarding LTx outcomes in this population.

The primary objective of this study was to describe Chinese WRLD patients who received LTx and their clinical outcomes. Idiopathic pulmonary fibrosis (IPF), like WRLD, is also a fibrotic lung disease and a major candidate for LTx [7]. Our secondary objective was to compare the outcomes of LTx between patients with WRLD and those with IPF.

## Methods

### Patients

This was a single-center retrospective cohort study. Recipients who underwent LTx between January 2015 and December 2020 at our hospital were retrospectively identified. The inclusion criterion was LTx for WRLD (including silicosis, coal workers’ pneumoconiosis, asbestosis, or other occupational lung diseases) or IPF. Considering that all the WRLD patients in our study were male, the female IPF recipients were excluded. Recipients who were younger than 18 years or older than 70 years were also excluded.

Organ donation and distribution were processed by the China Organ Transplant Response System using the Lung Allocation Score system for all study patients, which is the sole legitimate official organ allocation computer system in China. No organs from executed prisoners were used in this study. The study was approved by the Ethics Committee of the First Affiliated Hospital of Guangzhou Medical University (approval number: 2022 K-20). Written informed consent was waived due to the retrospective design of our study.

### Immunosuppressive treatment

Our center adopted a standardized immunosuppressive scheme, including an induction therapy consisting of rabbit antithymocyte globulin (r-ATG) and basiliximab, and a triple immunosuppression maintenance therapy consisting of a calcineurin inhibitor (cyclosporin A or tacrolimus), mycophenolate sodium or mycophenolate mofetil, and oral prednisolone. The decision of induction therapy was individualized for each patient considering the risk of rejection and infection. Graft surveillance included chest CT, bronchoscopy, pulmonary function tests, and exercise tolerance tests.

### Data collection

The following data were collected: recipient age, weight, and height at transplantation; recipient and donor cytomegalovirus serology status; baseline lung function tests including forced expiratory volume in 1 second (FEV1), forced vital capacity (FVC), and diffusing capacity of the lung for carbon dioxide (DLCO); 6-minute walk distance (6MWD); right heart catheterization values; induction therapy. All patients were followed up continuously until December 2021 or death.

### Clinical outcomes

The primary outcome was the cumulative survival rate at 30 days, 1 year, 3 years, and 5 years after LTx. The secondary outcomes included the length of intensive care unit (ICU) stay, length of hospital stay, pulmonary function, and exercise capacity after transplantation. Pulmonary function and exercise capacity were assessed by the best performance in the pulmonary functional tests and 6MWD within 1 year after transplantation.

### Statistical analysis

Statistical analysis was performed using SPSS software (version 19.0; IBM Corp., Armonk, NY, USA), and data were plotted using GraphPad Prism 5 software (GraphPad Software, Inc., San Diego, CA, USA). Continuous data with a normal distribution were expressed as mean ± standard deviation and compared by using the independent Student’s t-test. Those with a skewed distribution were expressed as median and interquartile range and compared by using the Wilcoxon rank-sum test. Categorical data were expressed as count and percentage and compared by using the Chi-square test. Kaplan-Meier survival curves were drawn and compared by the logrank test. A *P*-value less than 0.05 was considered statistically significant. Missing data were treated as blank cells in statistical analysis.

## Results

### Characteristics of the recipients

**Fig. 1:**
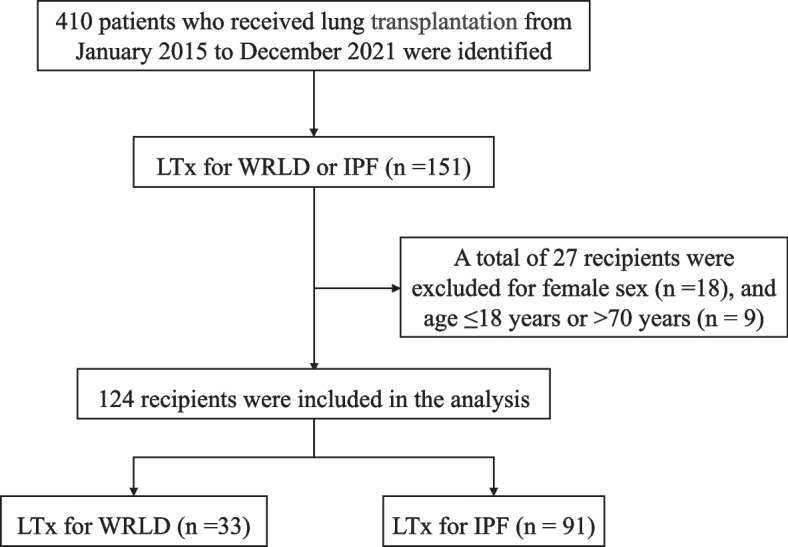
Flow chart of patient inclusion

The patient inclusion process is shown in Fig. 1. The final analysis included 33 WRLD patients and 91 IPF patients who received LTx. The baseline demographic and clinical characteristics of the included recipient are shown in Table 1. The 33 WRLD recipients consisted of 19 (57.6%) cases of silicosis caused by artificial stone, 8 (24.2%) cases of coal workers’ pneumoconiosis, 3 (9.1%) cases of asbestosis, and 3 (9.1%) cases of other WRLD caused by cement or mushroom spores. The WRLD patients and IPF patients represented 8.0% (33/410) and 22.2% (91/410) of the total LTx, respectively.

**Table 1:** Baseline demographic and clinical characteristics of the study patients

	WRLD (*n* = 33)	IPF (*n* = 91)	*P-*value
Age, years	52.5 ± 10.2	61.2 ± 6.7	< 0.001
Body mass index, kg/m^2^	18.9 ± 3.3	21.6 ± 3.3	0.96
Spontaneous pneumothorax, n (%)	17 (51.5)	2 (2.2)	< 0.001
FVC, %	40.71 ± 14.17	50.28 ± 14.14	0.02
FEV_1_, %	29.27 ± 13.14	52.68 ± 15.02	0.001
DLCO, %	28.9 ± 9.95	29.19 ± 12.66	0.07
6-minute walk distance, m	138.6 ± 58.7	150.4 ± 69.7	0.33
Left ventricular ejection fraction, %	70.0 ± 5.4	70.8 ± 6.0	0.51
Cardiac index, L/min/m^2^	2.9 ± 0.67	2.95 ± 0.87	0.88
Invasive mechanical ventilation, n (%)	4 (12.1)	11 (12.1)	0.99
Induction therapy, n (%)			0.83
Basiliximab	14 (42.4)	44 (48.4)	
r-ATG	13 (39.4)	45 (49.5)	
Type of lung transplantation, n (%)			0.024
Single	28 (84.8)	58 (63.7)	
Bilateral	5 (15.2)	33 (36.3)	

*DLCO* diffusing capacity of the lung for carbon monoxide, *FEV*_*1*_ forced expiratory volume in 1 s, *FVC* forced vital capacity, *WRLD* work-related lung disease, *IPF* idiopathic pulmonary fibrosis, *r-ATG* rabbit antithymocyte globulin

Before the LTx, 17 (51.5%) WRLD patients had at least one episode of pneumothorax, and 8 (24.2%) had recurrent pneumothorax. Most of them (14/17, 82.4%) had unilateral pneumothorax and 3 had bilateral pneumothorax. For these WRLD patients, recurrent and incurable pneumothorax were the major reason for LTx. The IPF patients had significantly better pre-transplant pulmonary function than the WRLD patients (Table 1). The two groups were generally similar in the 6MWD test, echocardiography, and respiratory support. Significantly more WRLD patients received single-lung transplantation than the IPF patients (84.8% vs. 63.7%, *P* = 0.024).

### Post-transplant outcomes and complications

At post-transplant 1 year, the WRLD group was comparable to the IPF group in terms of primary graft dysfunction and length of hospital and ICU stay. However, the WRLD patients had significantly more post-transplant hemorrhages than the IPF patient (Table 2). There were no significant differences in the best performance of 6MWD between the two groups at 1 year after transplantation, although the lung function results were generally better in the IPF group than in the WRLD group. However, these differences did not reach statistical significance when the two groups were stratified by single-lung or bilateral-lung transplantation (Table 3).

**Table 2:** Treatment outcomes and complications at post-transplant 1 year

	WRLD (*n* = 33)	IPF (*n* = 91)	*P-*value
Primary graft dysfunction, n (%)			0.23
Severe	15 (45.5)	38 (41.8)	
Non-severe	18 (54.6)	53 (58.2)	
Hemothorax, n (%)	10 (30.3)	4 (4.4)	< 0.001
Length of ICU stay, day, median (IQR)	10 (23–7)	9 (5–19)	0.18
Length of hospital stay, day, median (IQR)	31 (49–20)	26 (18–42)	0.24
Pulmonary function tests^a^			
FVC, %	68.5 ± 19.3	76.6 ± 17.5	0.04
FEV_1_, %	62.1 ± 18.6	79.4 ± 16.6	0.001
DLCO, %	61.0 ± 19.7	58.1 ± 17.5	0.57
6MWD, m	518 ± 144	505 ± 106	0.65

*DLCO* diffusing capacity of the lung for carbon monoxide, *FEV1* forced expiratory volume in 1 s, *FVC* forced vital capacity, *6MWD* 6-minute walk distance, *WRLD* work-related lung disease, *IPF* idiopathic pulmonary fibrosis, *IQR* interquartile range. ^a^Due to poor physical status and post-transplant complications, some patients did not have pulmonary function tests and the sample size was 26 in the WRLD group and 63 in the IPF group for FVC and FEV_1_, and 18 in the WRLD group and 46 in the IPF group for DLCO

**Table 3:** Lung function at post-transplant 1 year stratified by single- or bilateral-lung transplantation

	Sigle-lung transplantation			Bilateral-lung transplantation		
	WRLD (*n* = 28)	IPF (*n* = 58)	*P-*value	WRLD (*n* = 5)	IPF (*n* = 33)	*P-*value
FVC, %	72.6 ± 21.6	76.2 ± 17.5	0.99	88.4 ± 24.9	78.3 ± 17.6	0.67
FEV_1_, %	65.5 ± 19.7	76.14 ± 16.6	0.03	77.1 ± 23.9	79.4 ± 16.6	0.90
FVC, %	74.6 ± 10.5	86.4 ± 11.9	< 0.001	75.6 ± 9.27	86.1 ± 12.7	0.32
DLCO, %	60.2 ± 20.0	57.3 ± 18.0	0.63	57.7 ± 11.8	58.1 ± 17.5	0.39

*DLCO* diffusing capacity of the lung for carbon monoxide, *FEV1* forced expiratory volume in 1 s, *FVC* forced vital capacity

**Fig. 2:**
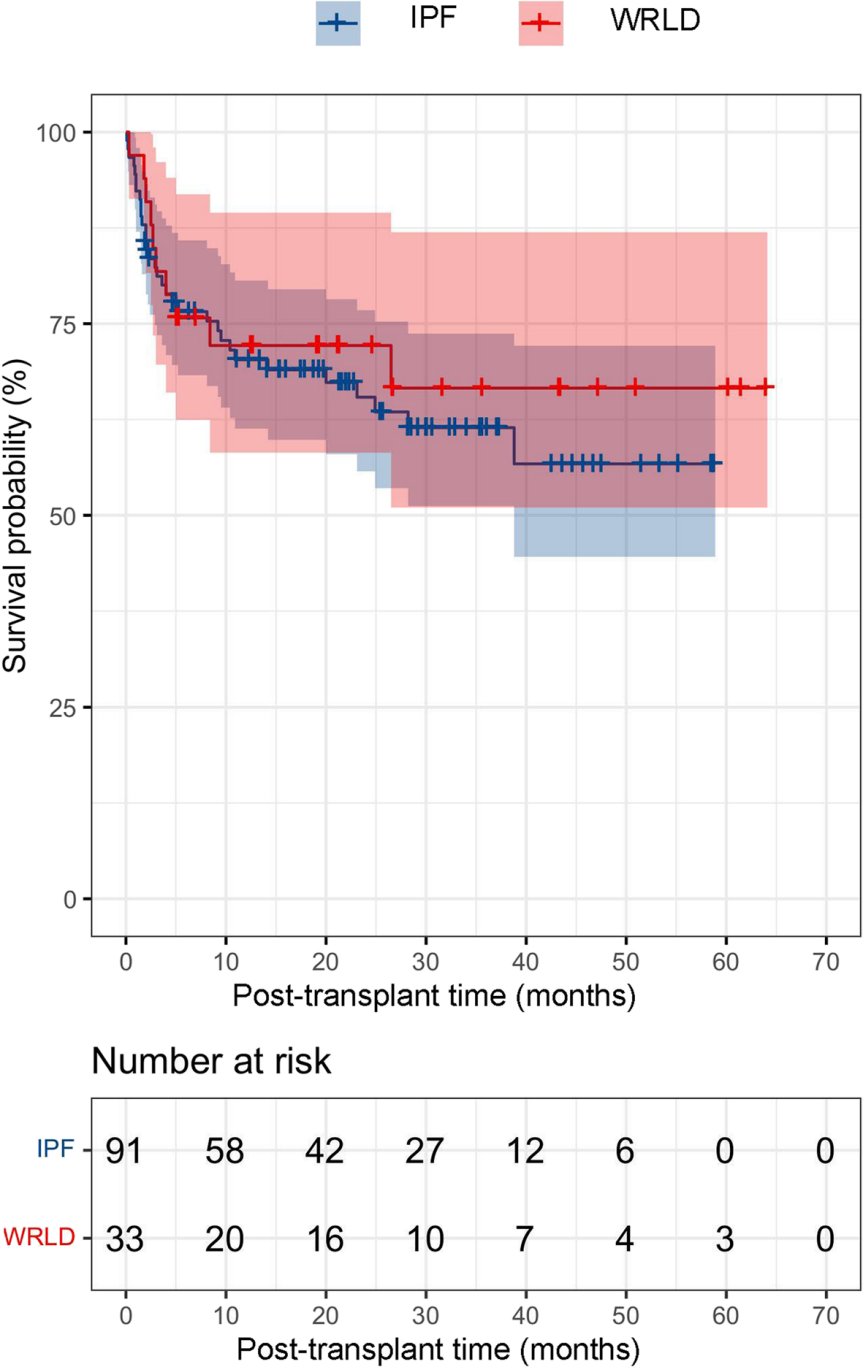
Kaplan-Meier survival curves comparing the post-transplant survival rates between the patients with work-related lung disease (WRLD) and those with idiopathic pulmonary fibrosis (IPF) (*P* = 0.67)

No significant difference was found in the cumulative survival rates between the WRLD group and the IPF group at post-transplant 1 year (75.8%, [95% confidence interval] CI: 61.1–90.5% vs. 87.9, 95% CI: 81.2–94.6%, *P* = 0.82), 3 years (66.6, 95% CI: 48.8–84.4% vs. 70.3, 95% CI: 60.7–79.9%, *P* = 0.83), and 5 years (66.6, 95% CI: 48.8–84.4% vs. 63.5, 95% CI: 52.7–74.2%, *P* = 0.67) (Fig. 2).

## Discussion

Our study for the first time analyzed the clinical outcomes and survival following LTx for WRLD in China. Artificial stone-associated silicosis accounted for most of our WRLD patients. Pneumothorax was the primary indication for LTx in the WRLD patients. WRLD and IPF patients had comparable post-transplant survival rate and complications.

Our study showed that the WRLD patients constituted 8.0% of the total patients who received LTx at our hospital, which is consistent with a previous number of 9.2% in a national report [8]. This result is unsurprising because our center ranks the second among the three major lung transplant centers in China. WRLD is prevalent worldwide and has maintained a high incidence in recent years [9]. However, the proportion of WRLD patients in LTx in our study is much higher than that reported in other parts of the world, which is between 0.5–1% [10, 11]. The underlying reason may be the high prevalence of WRLD in China, which is related to multiple factors such as poor work conditions, scarce of medical facility, rural area of residence, and low economic status of the workers [12, 13].

WRLD is often associated with extensive pleural adhesions, which may lead to severe bleeding during pleural dissection and lung transplantation. Therefore, significantly more patients in the WRLD group received single-lung transplantation to reduce the risk of major intraoperative bleeding. The WRLD patients had worse lung function compared to the IPF patients, and lung function tests showed that the former had both obstructive and restrictive pulmonary dysfunction. In addition, significantly more WRLD patients had spontaneous pneumothorax compared to the IPF patients, especially in those with silicosis. Also, multiple lung blebs were shown in thoracic CT images of these patients. Taken together, our study suggested that patients with WRLD suffered from impaired ventilation and restricted spirometry [14].

The association between silica exposure and emphysema has been noted [15]. Silicosis is an independent risk factor for pneumothorax [16]. Massive lung fibrosis can cause increased elastic recoil and collapse of the adjacent regions, leading to uneven expansion of the lung and pressure gradient, and finally rupture of the blebs [17]. More than half of our WRLD patients had spontaneous pneumothorax and even recurrent and non-curable pneumothorax. Pneumothorax was firstly managed with negative pressure drainage. Patients with poor response to this treatment were switched to pleurodesis induced by talcum powder. LTx was chosen as the final treatment if all these conservative treatments fail, and the patient develops respiratory failure. Thus, LTx might be the last resort for these patients. Our study suggested that spontaneous pneumothorax is a reasonable indication for LTx in patients with end-stage WRLD.

During the transplantation surgery and the immediate post-operative periods, severe chest wall bleeding or hemothorax were significantly more common in the WRLD group than the IPF group. No significant differences were noticed in other immediate post-operative outcomes including mechanical ventilator support, primary graft dysfunction, and ICU stay duration between the two groups. Long-term survival after transplantation was also similar between the WRLD and the IPF patients. Previous studies have also reported good clinical outcomes and survivability for WRLD patients after LTx [18–20]. In our study, the post-transplant lung function in the WRLD group was still slightly lower than the IPF group. This could be associated with the better baseline lung function and more double LTx in the IPF patients.

However, both groups of patients had significant improvement in 6MWD after transplantation, although no significant difference was noticed between the two groups. Therefore, LTx is a valid and effective treatment for end-stage WRLD.

Our study has some limitations. Firstly, although our center covers the south China, the findings should be cautiously interpreted due to the single-center study design and the small sample size. Secondly, the high 5-year mortality led to significantly less survival patients, which may bias our results. Thirdly, the younger age and the higher proportion of sing-lung transplantation in the WRLD group may have potentially improved survival and lung function in these patients. Fourthly, quite many of our patients did not have lung function tests due to poor physical status and post-transplant complications. The high proportion of missing data may reduce the statistical power of lung function.

## Conclusions

Pneumothorax is the primary indication for LTx in WRLD patients at our center. The post-transplant survival and exercise capacity were comparable between WRLD and IPF. Further investigation with larger sample sizes and multiple center design is needed to verify our findings.

## Data Availability

The data presented in this study are available on request from the corresponding author.

## Abbreviations

6MWD: 6-minute walk distance
CT: Computed tomography
DLCO: Diffusing capacity of the lung for carbon dioxide
FEV1: Forced expiratory volume in 1 second
FVC: Forced vital capacity
ICU: Intensive care unit
IPF: Idiopathic pulmonary fibrosis
LTx: Lung transplantation
WRLD: Work-related lung disease
